# Hybrid Human-Machine Interface for Gait Decoding Through Bayesian Fusion of EEG and EMG Classifiers

**DOI:** 10.3389/fnbot.2020.582728

**Published:** 2020-11-17

**Authors:** Stefano Tortora, Luca Tonin, Carmelo Chisari, Silvestro Micera, Emanuele Menegatti, Fiorenzo Artoni

**Affiliations:** ^1^Department of Information Engineering, University of Padova, Padova, Italy; ^2^Unit of Neurorehabilitation, Department of Medical Specialties, University Hospital of Pisa, Pisa, Italy; ^3^Department of Excellence in Robotics and AI Scuola Superiore Sant'Anna, The Biorobotics Institute, Pisa, Italy; ^4^Bertarelli Foundation Chair in Translational Neuroengineering, Center for Neuroprosthetics and Institute of Bioengineering, Lausanne, Switzerland; ^5^Functional Brain Mapping Laboratory, Department of Basic Neuroscience, Faculty of Medicine, University of Geneva, Geneva, Switzerland

**Keywords:** EEG, EMG, hybrid BCI, Bayesian inference, LSTM network, mobile brain/body imaging, locomotion

## Abstract

Despite the advances in the field of brain computer interfaces (BCI), the use of the sole electroencephalography (EEG) signal to control walking rehabilitation devices is currently not viable in clinical settings, due to its unreliability. Hybrid interfaces (hHMIs) represent a very recent solution to enhance the performance of single-signal approaches. These are classification approaches that combine multiple human-machine interfaces, normally including at least one BCI with other biosignals, such as the electromyography (EMG). However, their use for the decoding of gait activity is still limited. In this work, we propose and evaluate a hybrid human-machine interface (hHMI) to decode walking phases of both legs from the Bayesian fusion of EEG and EMG signals. The proposed hHMI significantly outperforms its single-signal counterparts, by providing high and stable performance even when the reliability of the muscular activity is compromised temporarily (e.g., fatigue) or permanently (e.g., weakness). Indeed, the hybrid approach shows a smooth degradation of classification performance after temporary EMG alteration, with more than 75% of accuracy at 30% of EMG amplitude, with respect to the EMG classifier whose performance decreases below 60% of accuracy. Moreover, the fusion of EEG and EMG information helps keeping a stable recognition rate of each gait phase of more than 80% independently on the permanent level of EMG degradation. From our study and findings from the literature, we suggest that the use of hybrid interfaces may be the key to enhance the usability of technologies restoring or assisting the locomotion on a wider population of patients in clinical applications and outside the laboratory environment.

## 1. Introduction

Neurological diseases after brain or spinal cord lesions are one of the major causes of locomotion impairments, leading to functional disability and loss of independence in daily-living activities for patients (Barbeau et al., 1999; Sarti et al., 2000). To reach better independence, gait rehabilitation is key to regain walking capacity and the recent advances in bio-robotics and neuroprostheses have provided many technological solutions to restore locomotion. Functional improvements have been reported in stroke survivors (Chisari et al., 2015; Morone et al., 2018) and spinal cord injury (SCI) patients (Kolakowsky-Hayner et al., 2013; Donati et al., 2016; Shokur et al., 2018) after robot-aided training with a lower limb exoskeleton. Recently, epidural electrical stimulation (EES) has shown very promising performance in restoring locomotion after spinal lesion (Harkema et al., 2011; Wenger et al., 2014; Wagner et al., 2018). In this context, it is of paramount importance to develop advanced human-machine interfaces to enhance the interaction between the user and the rehabilitation device and thus, his active involvement, and maximizing the rehabilitation outcome (Edgerton et al., 2008).

Electromyography (EMG) is certainly one of the most used techniques to control rehabilitation and assistive devices, particularly in robotic applications. EMG has been already successfully used to decode gait activity (Meng et al., 2010; Joshi et al., 2013; Li et al., 2016; Luo et al., 2019), and to control exoskeletons (Fleischer et al., 2005; Ferris and Lewis, 2009; Takahashi et al., 2015) or neuroprostheses based on functional electrical stimulation (FES) (Graupe et al., 1982; Dutta et al., 2008). However, the usability of EMG-based control strategies strongly depends on the user's residual muscular functions. If the EMG activity is highly affected by the limb's paresis following a neurological disease, the decoding of user's movement intention could be compromised by muscle weakness and early fatigue, making the use of these assistive technologies unfeasible on severely impaired patients (Millán et al., 2010). For this reason, brain computer interface (BCI) technologies based on non-invasive Electroencephalography (EEG) have been introduced to decode movement intention from brain electrical signals, even in absence of any muscular activity. Previous works have proven the feasibility of predicting motion intention with BCI to detect sit-to-stand and stand-to-sit movement (Chaisaen et al., 2020), to trigger lower limb exoskeletons (Kilicarslan et al., 2013; Lee et al., 2017), but also to decode walking patterns from EEG signals (Presacco et al., 2011; Nakagome et al., 2020; Tortora et al., 2020). However, interfaces based on EEG signals alone are not reliable enough for most clinical applications and to control advanced neurorobotics devices yet due to their low reliability, low accuracy and low informative content (Vaughan et al., 1996; Wolpaw et al., 2000). These issues have strongly limited the diffusion of these technologies on a wider population of patients and for applications outside a controlled laboratory environment.

In this study, we propose a hybrid human-machine interface (hHMI) fusing the motion information coming from the brain signals with the information coming from the natural muscular activation with the purpose of decoding walking activity. The aim of the fusion method proposed in this study is to enhance the reliability of the human-machine interface by integrating the outputs of an EEG and an EMG classifiers using principles of Bayesian inference (Ruta and Gabrys, 2000). The measurement of a human-machine interface reliability is a current challenging topic, since it can be analyzed from a 2-fold point-of-view: from the adaptation of the user to the interface or from the adaptation of the interface to the user, measured as the performance of the machine learning system to changes of the input signals (e.g., session-to-session variability, non-stationarity of the driving signals) (Perdikis et al., 2018). In this work, we evaluated the proposed hHMI from the latter point-of-view. By weighing the contribution of each classifier based on the confidence of its prediction, the proposed hybrid interface enhance the overall decoding performance, particularly when the reliability of muscular signals is affected, either temporary (e.g., muscle fatigue) or permanently (e.g., muscle weakness). To evaluate this capacity, the performance of the proposed hybrid approach was compared to the single-signal approaches also with respect to simulated conditions of EMG signal alteration. These evaluations have been carried out simulating an online usage of the hHMI in order to exploit this interface to control a lower limb exoskeleton or a neuroprosthesis in future work.

### 1.1. Related Work

Recent attempts to overcome the limitations of conventional BCI systems have brought forward approaches combining multiple human-machine interfaces, including at least one BCI integrated with other BCIs or other biosignals (e.g., EMG) or input devices (e.g., joysticks) (Müller-Putz et al., 2015). This combination may happen at different levels of the processing flow: at the features level (Li et al., 2017), at the decision-making level (Leeb et al., 2011), or at the control level (Kiguchi et al., 2013), by having each channel controlling a different part or behavior of the assistive device. These hybrid solutions often rely on a fusion module integrating the outputs of multiple classifiers driven by different brain features (Millán et al., 2010). For example, Yin et al. (2015) proposed a hybrid BCI integrating the classification of a P300 BCI and a steady-state visual evoked potential (SSVEP) BCI with a maximum probability estimation (MPE) fusion approach. The same research group implemented also a bimodal P300 BCI approach that simultaneously employs auditory and tactile stimuli (Yin et al., 2016). In Ferrez and Millán (2008) the reliability of a motor imagery BCI is improved by discarding erroneous commands if they generate an error-related potential (ErrP) right after the classification.

Many patients present residual motor functions that may be used as additional control inputs in parallel with the BCI. For example, in Rohm et al. (2011) the remaining shoulder function of a SCI patient is exploited to control an upper-limb neuroprosthesis, while the motor imagery of hand movement is used to toggle the different control modalities of the assistive device. In this category of hHMIs, solutions based on EEG and EMG signals may increase the reliability of the interface with respect to both signals alone in upper limb experiments (Carlson et al., 2013; Chowdhury et al., 2017). In particular, the additional analysis of EEG signals in parallel with EMG activity have shown to improve the detection of motion intention in patients with compromised muscular functions (Bhagat et al., 2016; Lóopez-Larraz et al., 2018). Leeb et al. (2011) showed the benefit of a multimodal EEG-EMG approach to classify between movement of right and left hands independently of the level of muscular fatigue. In lower limb applications, EEG and EMG signals have been primarily used to monitor the user's condition while walking, for example to measure the workload (Matthews et al., 2008) or predict the risk of falling (Annese and De Venuto, 2015). In walking classification applications, EEG and EMG signals are rarely used simultaneously, but rather as a cascade of classifiers. In Du et al. (2012), an EEG-based interface is used to control a lower limb exoskeleton by detecting walking direction, while walking patterns are decoded from the EMG signal alone. In Li et al. (2019), the intention to take a step is decoded from the EEG signals only, while the EMG signals from the upper limbs are exploited to determine the step height while climbing stairs. In Sherwani and Kumar (2016), the predictions of an EEG and an EMG classifier are fused to predict sit-to-stand and stand-to-sit tasks. On the other hand, the development of Mobile Brain/Body Imaging (MoBI) frameworks (Gennaro and De Bruin, 2018), allowing the simultaneous acquisition of EEG and EMG signals during walking, enabled scientists to deepen the understanding of both cortical and muscular control of gait patterns (Storzer et al., 2016; Artoni et al., 2017).

Despite these findings however, to the best of our knowledge, EEG and EMG have never been used simultaneously to decode walking activity. To this aim, this paper contributes to the field by proposing and evaluating a hHMI decoding gait events from the fusion of brain and muscular activity. In particular, two deep neural networks are trained with EEG and EMG signals, respectively, in order to predict step onset (i.e., swing phase) and offset (i.e., stance phase) of right and left legs. Finally, their predictions are integrated at the decision-making level using a Bayesian belief fusion method, based on the uncertainty of each classifier in predicting that the subject is in a certain gait phase. We believe that the proposed solution may guarantee more stable performance of the human-machine interface controlling the walking restoration device, by adapting the fusion parameters to the changes of input signals reliability in clinical and daily-living applications.

## 2. Materials and Methods

### 2.1. Experimental Setup and Data Acquisition

The hHMI proposed and described in this paper was validated on a pre-recorded dataset containing one session of EEG and EMG recordings from eleven healthy subjects (mean age 30 ± 4 years) while walking. During each session, the subject was asked to walk on a treadmill at 2.5 and 3.5 km/h, for 10 min for each walking speed, thus 20 min overall. EEG data were recorded with a signal pre-amplifying active electrode cap (actiCAP, Brain Products GmbH, Germany) and a 64-channel EEG amplifier (SD MRI, Micromed S.p.A., Italy) with a sampling rate of 2,048 Hz. EMG data were simultaneously recorded with a wireless EMG system (BTS Free EMG 300) at a sampling rate of 1,000 Hz. EMG electrodes were placed on Tibialis Anterior (TA), Vastus Medialis (VM) and Biceps Femoris (BF) of each leg, according to SENIAM guidelines (www.seniam.org). Swing and stance events were identified from the signals acquired with four foot-switches positioned under the shoes of each subject, two under the heel and two under the toes. Data from the foot-switches were wirelessly acquired through the EMG system with a sampling frequency of 1,000 Hz. EEG, EMG and foot-switches data were all resampled to 1,024 Hz. This dataset was previously published and was acquired within the MoBI framework presented in Artoni et al. (2018), more details on the experimental protocol and data acquisition can be found in Artoni et al. (2017) and Tortora et al. (2020). All subjects provided informed consent prior to participation in this study. This study was carried out in accordance with the declaration of Helsinki.

### 2.2. Data Processing

**Figure 1:**
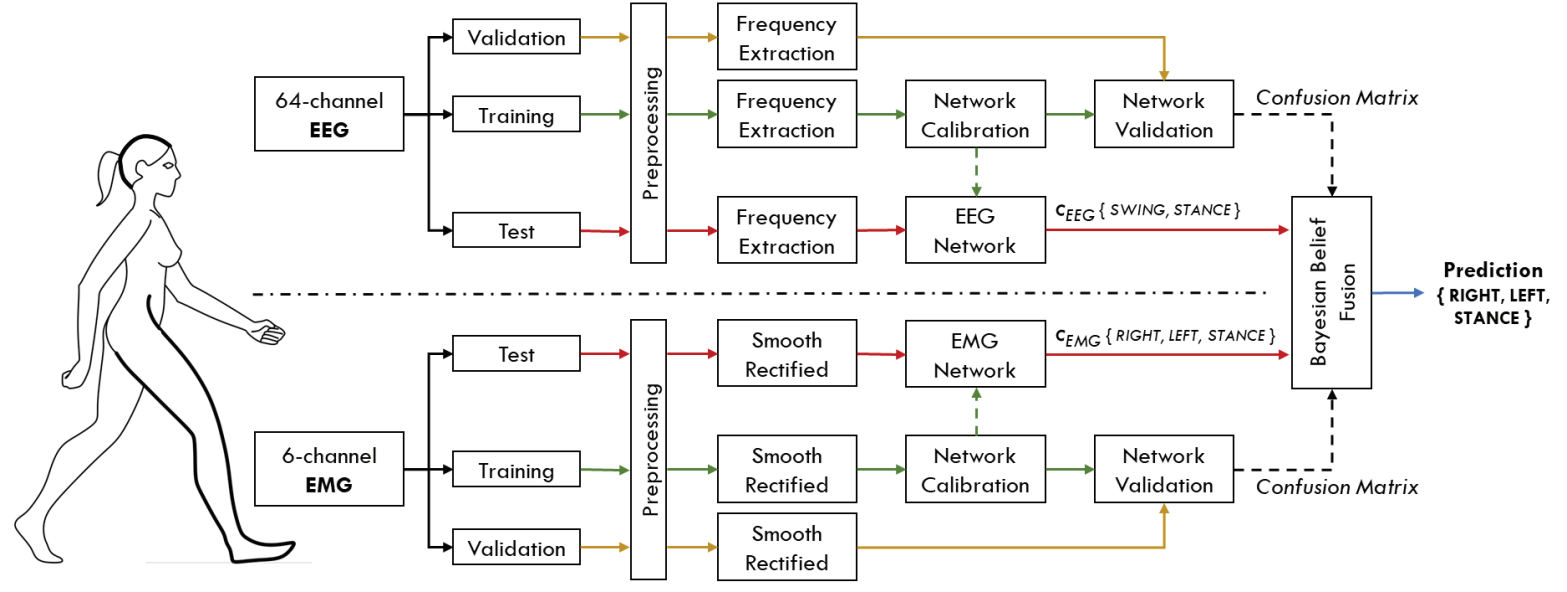
Schematic representation of the proposed hybrid human-machine interface. Brain activity is registered with a 64-channel EEG system, while three lower limb muscles per leg are simultaneously recorded within the same acquisition set up. Each subject's dataset is separated into three distinct groups of data, namely training, validation and test set. Data from the training set (green arrows) are used to calibrate the parameters of the two classification networks. The performance of each network, in terms of confusion matrices, are evaluated on the validation set (yellow arrows) for the EEG and EMG signals independently. These performance measures are then used to guide the Bayesian fusion method of the classifier's output (*c*_*EEG*_, *c*_*EMG*_), to predict gait events on the data coming from the test set (red arrows).

This section describes the processing procedures applied to EEG and EMG signals before being used as input to their respective classifiers. In order to simulate an online condition, the dataset of each subject was divided into three groups: a training set (60% of the dataset, about 12 min of walking), a validation set (15% of the dataset, about 3 min of walking) and a test set (25% of the dataset, about 5 min of walking). Networks were trained with data of the training set only, while the parameters for the Bayesian belief fusion were obtained from the validation set. Once the Bayesian fusion parameters have been estimated, the networks were re-trained with the ensemble of training and validation set, in order to maximize the data available for classifier's learning. Finally, the overall performance were evaluated on the data of the test set. All the processing procedures applied to the test set are compatible with an online usage of the interface. A representation of the proposed hHMI is schematically shown in Figure 1.

#### 2.2.1. EEG Processing

Generally, EEG data are contaminated by many external sources (Gwin et al., 2010), particularly gait-locked artifacts overlapped with brain signals (Kline et al., 2015). Thus, preprocessing procedures are necessary to clean the EEG dataset from non-brain activity. To this aim, we applied a previously validated processing procedure, described in detail in Tortora et al. (2020). Different processing stages were applied on the three groups of data. Training and validation set were preprocessed with a two-step offline preprocessing, following a procedure previously described and validated in Artoni et al. (2017), to ensure that the classifier learning and the Bayesian fusion parameters were not influenced by any source of noise generated during the locomotion. On the other hand, a more conservative preprocessing was applied on the test set to guarantee its usability in an online application:

- *Preprocessing Step I*: raw EEG data were high-pass filtered with a zero-phase 24th-order Chebyshev type II filter above 1 Hz and low-pass filtered with a zero-phase 71th-order Chebyshev type II filter below 98 Hz. Power line interference were removed with a comb notch filter at 50 Hz. Manual and automatic procedures were used to reject corrupted EEG channels and epochs containing high-amplitude (above 100 μV) and irregular artifacts (Artoni et al., 2012). A Common Average Reference (CAR) filter was then applied to the remaining channels.
- *Preprocessing Step II*: raw EEG data were high-pass filtered with a zero-phase 24th-order Chebyshev type II filter above 1.5 Hz and low-pass filtered with a zero-phase 71th-order Chebyshev type II filter below 48 Hz. The data were then resampled to 512 Hz. The channels removed during *Step I* were also removed in this step. A CAR filter was then applied to the remaining channels. Non-stereotypical artifacts (e.g., movement artifacts related to cable displacement) were removed through Artifacts Subspace Reconstruction (ASR) (Kothe and Jung, 2016; Chang et al., 2018). Then, the EEG signals were decomposed into maximally independent processes through an infomax Reliable Independent Component Analysis (RELICA) (Artoni et al., 2014). The ICA weights were applied to the EEG signals coming from *Step I*, projecting the data into the independent components domain. Components that belong to stereotypical artifacts (e.g., neck muscles, eye movement) were rejected by backprojecting the EEG signals to the original domain using only the components related to brain activity (Artoni et al., 2017).

**Figure 2:**
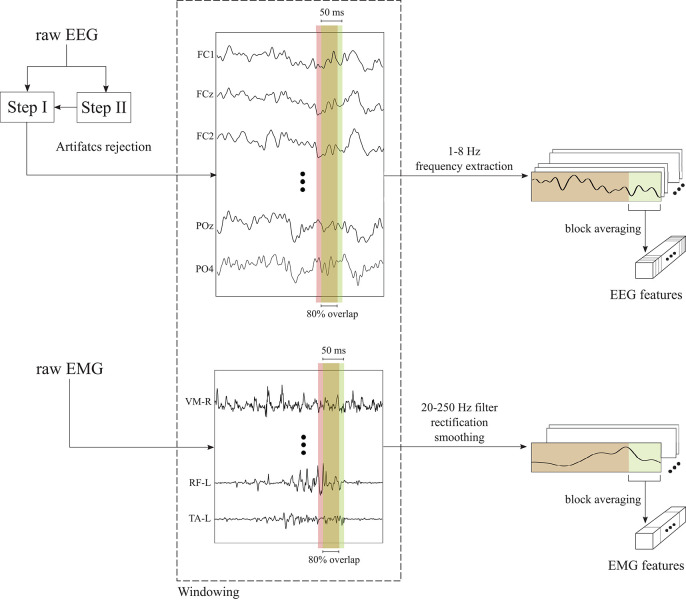
Schematic representation of the preprocessing procedures applied to the EEG and EMG signals. EEG signals are firstly preprocessed with a two-step preprocessing to reject motion artifacts. Then, EEG and EMG time series are segmented in time windows of 50 ms with 80% overlap. After the windowed signals are further processed, the non-overlapping samples of each window (i.e., the last 20%) are block averaged in order to obtain a single EEG or EMG features per channel.

The two-step preprocessing was necessary to apply the Artifacts Subspace Reconstruction (ASR) only on the EEG data used for the estimation of the independent components (*Step II*) to enhance their reliability (Artoni et al., 2014), while the classifier was trained on the data from *Step I*, since ASR may remove also useful brain activity (Artoni et al., 2017). Channels rejected during these offline preprocessing stages were also removed from the test set. Data belonging to the training, validation and test set were segmented with a rectangular time window of 50 ms with 80% overlap, to achieve a classification at a rate of 1/((1 − 0.8) * 0.050*s*) = 100 *Hz*. A Common Average Reference (CAR) filter is applied to the chunk of EEG data inside each window and the δ-θ band (1-8 Hz) was extracted with a 4th-order zero-lag Butterworth filter. This frequency range was selected since it provided the best performance in a previous study (Tortora et al., 2020) and it is in line with the literature showing that meaningful changes during walking occur principally at frequencies below 10 Hz (Gwin et al., 2010, 2011). Data were normalized to have zero mean and unit standard deviation inside each window. Finally, a single-feature per channel for each window for classification was obtained by taking the block average of the processed EEG signals in the non-overlapping portion (i.e., the last 20%) of each window. The EEG preprocessing is schematically shown in Figure 2 (top).

#### 2.2.2. EMG Processing

EMG data of training, validation and test set were processed following the same procedures. Firstly, the EMG signals were segmented with a rectangular time window of 50 ms with 80% overlap, as the EEG signals. Inside each window, EMG signals were band-pass filtered in the range 10-250 Hz with a 8th-order zero-lag Butterworth filter, in order to remove low-frequency motion artifacts and high-frequency electrical noise. Then, the filtered EMG signals were rectified and smoothed using a 4th-order zero-lag Butterworth low-pass filter with cut-off frequency at 6 Hz. As for the EEG signals, a single-feature per channel for each window was obtained by taking the block average of the processed EMG signals in the non-overlapping portion (i.e., the last 20%) of each window. The EMG preprocessing is schematically shown in Figure 2 (bottom).

### 2.3. EEG and EMG Long-Short Term Memory (LSTM) Networks

Gait decoding classifiers were implemented as a deep neural network with recurrent layers, namely Recurrent Neural Network (RNN), whose output is a non-linear function of the current input (i.e., EEG or EMG channels) and its outputs at the previous time steps (Williams and Zipser, 1989). This characteristic provides the network with memory allowing to encode time dependency within the classification framework (Elman, 1990). Herein, we considered a particular implementation of a RNN, based on Long Short-Term Memory (LSTM) layers. LSTM networks are recurrent networks capable of learning long-term dependencies in time series without suffering from the vanishing gradient problem (Pascanu et al., 2013). This network was selected since it has already shown to be very effective in decoding gait events from either EEG (Tortora et al., 2020) and EMG (Luo et al., 2019) signals. The implementation of a LSTM network can be found in Hochreiter and Schmidhuber (1997), Gers et al. (1999), and Tortora et al. (2020) and it is beyond the scope of the paper.

**Figure 3:**
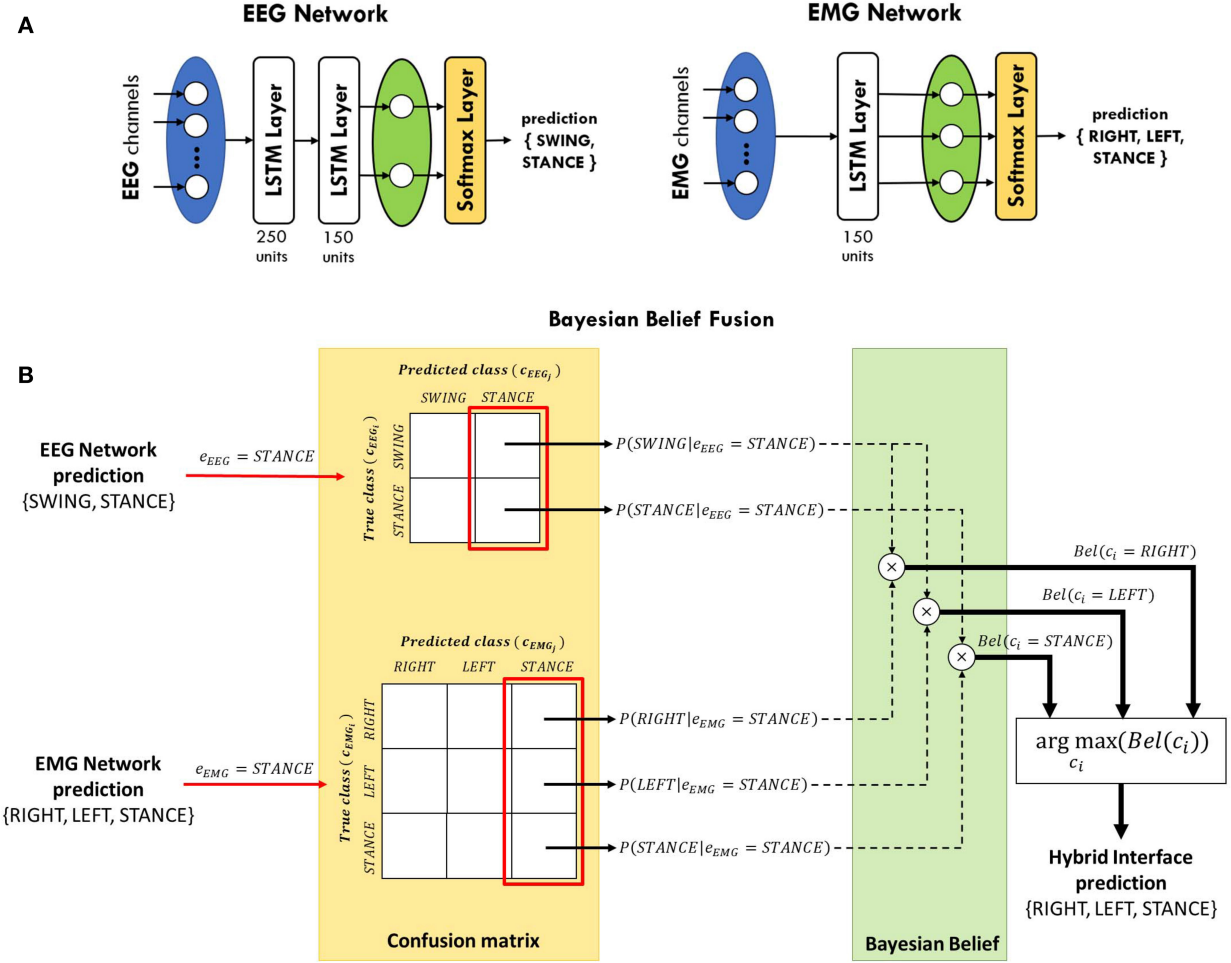
**(A)** LSTM networks' architecture for gait decoding from EEG and EMG signals. The EEG network consists of two LSTM layers, with 250 and 150 units, respectively. The output of the last layer is then processed by a fully-connected layer (green oval) and a softmax layer in order to classify between swing and stance phases. The EMG network contains only one layer with 150 units and it classifies between right swing, left swing, and stance phases. **(B)** Schematic diagram of Bayesian belief fusion of EEG and EMG networks classification, for a sample scenario in which both EEG and EMG networks predict the STANCE class. The output of each classifier (*e*_*EEG*_, *e*_*EMG*_) is used to determine the conditional probability of each true class (*c*_*EEG*_*i*__, *c*_*EMG*_*i*__) given the predicted class (*c*_*EEG*_*j*__, *c*_*EMG*_*j*__) from the confusion matrix of each classifier, estimated during the validation phase. The Bayesian belief *Bel*(*c*_*i*_) of each class *c*_*i*_ is computed by multiplication of the conditional probabilities of the EEG and EMG classifiers. The class predicted by the hybrid interface is the one maximizing the Bayesian belief.

In this study, two separated LSTM networks, running in parallel, were implemented to decode gait events from EEG and EMG, respectively, and their predictions are subsequently integrated (see section 2.4). Each network is provided with a sequence input layer with size equal to the number of channels used for classification. Then, channels' signals are processed by one or more LSTM layers to extract information from each time series that is correlated with the gait events to be classified. Channels' information is integrated in the fully-connected layer, with size equal to the number of classes to be identified, and the posterior probability of each class is provided in output by the softmax layer (see Figure 3A). The EEG network was trained to classify the swing phase of either right and left leg (*SWING*) against the double-stance phase (*STANCE*). On the other hand, the EMG network was trained to solve a 3-class classification problem, detecting the swing phase of the right leg (*RIGHT*), of the left leg (*LEFT*), and the double-stance phase (*STANCE*). An EEG and an EMG network were trained for each subject independently with the data from the training set only. The networks' architectures (i.e., number of LSTM layers, number of units per layer), shown in Figure 3A, were empirically defined for EEG and EMG signals separately based on the performance on the validation set, averaged across subjects, and on previous studies in literature (Craik et al., 2019; Luo et al., 2019; Roy et al., 2019; Tortora et al., 2020).

### 2.4. Bayesian Belief Fusion

The reliability of a classifier can be comprehensively described by means of confusion matrix, measuring the errors distribution of each network over the classes:

(1) $$\begin{array}{l} CM_{k}=\left[\begin{array}{ccccc} n_{11}^{k} & \ldots & n_{1j}^{k} & \ldots & n_{1M}^{k}\\ \ldots & \ldots & \ldots & \ldots & \ldots \\ n_{i1}^{k} & \ldots & n_{ij}^{k} & \ldots & n_{iM}^{k}\\ \ldots & \ldots & \ldots & \ldots & \ldots \\ n_{M1}^{k} & \ldots & n_{Mj}^{k} & \ldots & n_{MM}^{k} \end{array}\right] \end{array}$$

where each row corresponds to the class (i.e., *c*_1_, …, *c*_*M*_) the samples truly belong, as defined by the foot-switch signals, and each column to the class which the samples are assigned to. Thus, $n_{ij}^{k}$ represents the number of samples belonging to the *i* − *th* class that were predicted as the *j* − *th* class by the *k* − *th* classifier. Given *K* classifiers predicting among *M* classes, the general belief measure of correct assignment can be computed for each class *i* by combining the estimation *e*_*k*_ of each classifier as Ruta and Gabrys (2000):

(2) $$\begin{array}{l} Bel\left(c_{i}\right)=P\left(c_{i}\right)\frac{\prod_{k=1}^{K}P\left(c_{i}|e_{k}=c_{j}\right)}{\prod_{k=1}^{K}P\left(c_{i}\right)}\;with\;P\left(c_{i}|e_{k}=c_{j}\right)\\ \;=\frac{n_{ij}^{k}}{\sum_{i=1}^{M}n_{ij}^{k}},i=1,\ldots ,M;j=1,\ldots ,M \end{array}$$

where *P*(*c*_*i*_) is the prior probability of class *i* and *P*(*c*_*i*_|*e*_*k*_ = *c*_*j*_) is the probability that the true class is *i* when the *k* − *th* classifier outputs class *j*. In our case, *K* = 2 since we have one EEG network and one EMG network. However, the two networks do not provide the same number of classes *M*, as explained in section 2.3. Thus, we adapted (2) to our scenario as follow:

(3) $$\begin{array}{ll} Bel\left(c_{i}\right)=\frac{\prod_{k=\left\{EEG,EMG\right\}}P\left(c_{k_{i}}|e_{k}=c_{k_{j}}\right)}{P\left(c_{i}\right)}, & with:\\ c_{EEG_{i}}=\left\{SWING,SWING,STANCE\right\};\\ c_{EEG_{j}}=\left\{SWING,SWING,STANCE\right\};\\ c_{EMG_{i}}=\left\{RIGHT,LEFT,STANCE\right\};\\ c_{EMG_{j}}=\left\{RIGHT,LEFT,STANCE\right\};\\ \;c_{i}=\left\{RIGHT,LEFT,STANCE\right\};\\ \;i,j=1,2,3 \end{array}$$

In *c*_*EEG*_*i*__ and in *c*_*EEG*_*j*__ the SWING class is repeated twice since the EEG network provides the same contribution for either the right swing (RIGHT) and left swing (LEFT) events classified by the EMG network. In short, the EEG network detects if the subject is taking a step forward, regardless of which leg is moving, while only the EMG network is in charge of discriminating between the two legs. The class *c*_*i*_ with the highest belief *Bel*(*c*_*i*_) is chosen as the final decision of the classification framework. A schematic representation of the Bayesian belief fusion applied in this work is shown in Figure 3B for a sample scenario in which both EEG and EMG networks predict the *STANCE* class. Nevertheless, the same considerations could be applied when the two networks predict different classes. The conditional probabilities *P*(*c*_*k*_*i*__|*e*_*k*_ = *c*_*k*_*j*__) of each classifier were obtained on the validation set and the performance of the fusion method was evaluated on the test set. Since the walking speed is not known to the classifier, we used the same prior probability for all the classes.

### 2.5. Experimental Validation

**Figure 4:**
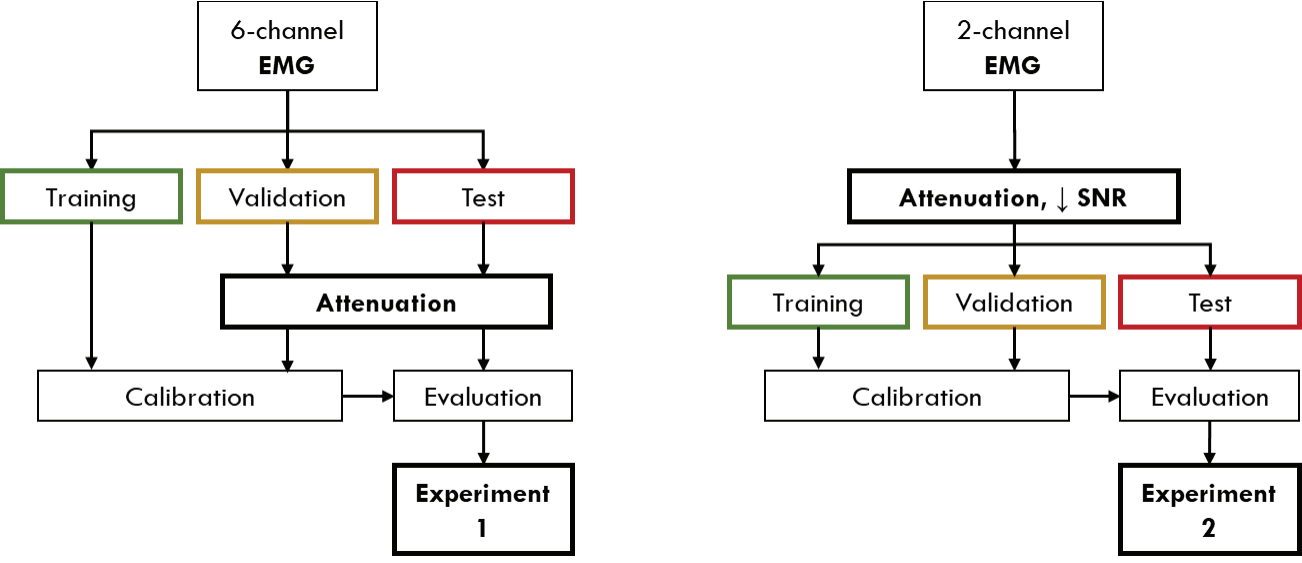
The processing pipeline applied to the EMG signals in order to validate the proposed approach in presence of temporary (Experiment 1) or permanent (Experiment 2) conditions of EMG alteration. In the first condition, signal attenuation is applied only to the validation and test set, while the original training set is used to calibrate the classifier. In the second experiment, signal attenuation and reduced signal-to-noise ratio (SNR) are applied on the entire EMG dataset, in order to simulate a steady condition of impairment.

It can be easily expected that the use of the sole EMG signal is enough to achieve an accurate decoding of gait phases in conditions where the nervous and/or muscoloskeletal systems are not impaired (Luo et al., 2019), with respect to what could be achieved by an EEG-based classifier, thanks to its higher correlation with the movement. However, in order to evaluate the proposed hHMI with the available dataset, containing EEG and EMG of healthy subjects only (see section 2.1), we simulated two different conditions in which the reliability of the EMG is compromised, either temporary (section 2.5.1) or permanently (section 2.5.2). These simulations were obtained by altering the distribution of EMG signals' amplitude in training, validation and test set, as shown in Figure 4 and explained in the following sections. Before the experiments, subjects *s02* and *s04* were excluded from the analysis since their EEG networks did not achieve performance above the level of significance (i.e., chance level of 50%). Since the performance and analysis of the EEG network on this dataset, including subjects *s02* and *s04*, have been previously published (Tortora et al., 2020), we decided to focus the paper on the integration of EEG and EMG signals to deal with the degradation of muscular activity, excluding those subjects for which the use of the EEG network would have just worsened the performance independently on the level of EMG alteration.

#### 2.5.1. Experiment 1: Temporary EMG Alteration

We evaluated the proposed hHMI in presence of a temporary alteration of the EMG signal. In this experimental condition, we assumed that only the EMG data during the human-machine interface usage were compromised, e.g., by fatigued-induced muscular changes, while muscles activity was unaltered during data collection for network training. In order to simulate this condition in the current study, on the one hand we applied a linear attenuation of EMG amplitude, ranging from 10 to 90%, to the validation and test set of each subject (Leeb et al., 2011). On the other hand, the EMG network parameters were calibrated with the original EMG signals.

#### 2.5.2. Experiment 2: Permanent EMG Alteration

Subjects who suffer of a neuromuscular disease or trauma, like stroke survivors or SCI patients, are often primarily impaired by weakness for voluntary muscle contraction due to a permanent paresis (Thomas et al., 1997; Kamper et al., 2006). This impairment is often reflected in a permanent alteration of the EMG activity. In order to simulate this condition, we considered to have available only one proximal lower limb muscle (i.e., VM) for each leg. Moreover, we applied to the whole EMG dataset a linear attenuation of 70% of the amplitude (van der Krogt et al., 2012) and we added a Gaussian noise (Li et al., 2007; Xu et al., 2012), with standard deviation determined to have different signal-to-noise ratio (SNR) levels—defined as the ratio of the signal power (i.e., the EMG activity informative for the movement) to the noise power, thus the background EMG activity—of 10, 3, 1.5, 1, 0.5, 0.1 dB, respectively. This procedure was applied on training, validation and test set of each subject, since we assumed the impairment to not change between classifier calibration and evaluation.

## 3. Results

**Figure 5:**
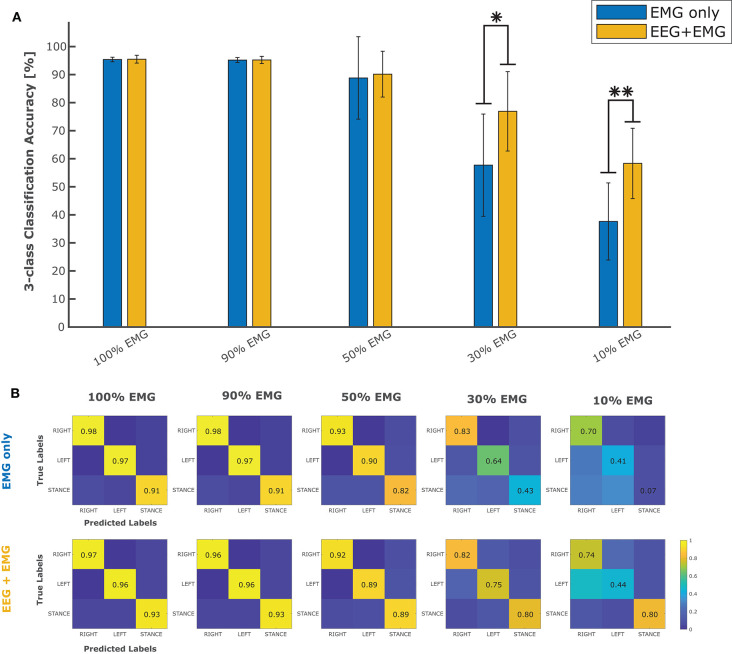
**(A)** Average classification accuracy in five different conditions (100, 90, 50, 30, and 10% of EMG signal amplitude) using only the EMG signals (blue bars) to classify between right swing, left swing and stance phases, with respect to the performance achieved by the use of EEG and EMG signals together (yellow bars). **(B)** Confusion matrices obtained on the test set by the classifier using only the EMG signals and the hybrid (EEG + EMG) approach at different levels of EMG amplitude. ^*^p < 0.05; ^**^p < 0.01.

**Figure 6:**
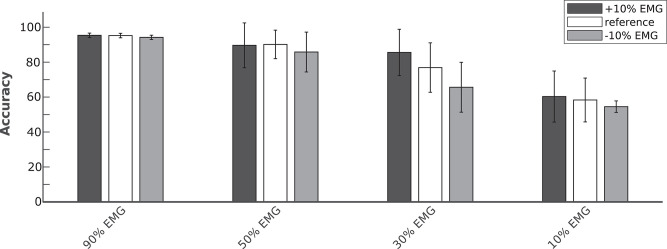
Average classification accuracy of the hybrid approach at four levels of temporary EMG alteration (90, 50, 30, and 10% EMG amplitude). The Bayesian fusion method provides comparable performance when the level of alteration is precisely known a priori, meaning the same attenuation is applied to both validation and test set (white bars), with respect to when the test set has a different attenuation level of +10% (dark gray bars) or −10% (light gray bars) of EMG signal amplitude than the validation set.

In order to evaluate the performance of the proposed hybrid approach, that integrates the information from the brain signals to support the compromised muscular activity, in the experiments we evaluated the performance of the single modality EMG network alone with respect to the performance obtained by the fusion method in detecting right swing, left swing and double-stance phases. Figure 5A shows the results for the five different conditions of the Experiment 1 (i.e., 100, 90, 50, 30, 10% of EMG signals amplitude). Performance is reported as the sample-by-sample accuracy over the whole test set, averaged across all the subjects. A statistically significant impact of classifier type (i.e., EMG only or EEG+EMG) and level of attenuation was found (Friedman test, *p* < 0.05). As shown in Table 1, the EMG network alone achieved an accuracy of 95% for almost all the subjects when no attenuation had been applied or at 10% of attenuation. Then a drop of EMG network performance could be seen in relation with the increase in EMG amplitude attenuation (e.g., higher level of fatigue) reaching less than 90% of accuracy at 50% of attenuation. A statistically significant decrease of performance has been found at 70 and 90% of attenuation up to less than 40% of accuracy (Bonferroni *post-hoc*, *p* < 0.05). Figure 5B better highlights the performance achieved in the Experiment 1 for each gait event to be identified. It can be seen that the reduction of the classification performance of the EMG network is mainly related to the difficulty in detecting the events related to the stance phase, from 91% of correctly detected samples to less than the chance level with 30 and 10% of EMG signals amplitude. On the other hand, with the proposed hHMI the degradation of the performance was remarkably lower, with a statistically significant improvement of about 20% of accuracy at 70% (Bonferroni *post-hoc*, *p* < 0.05) and 90% (Bonferroni *post-hoc*, *p* < 0.01) of attenuation. In particular, the stance detection rate was always above 80% even at the highest levels of EMG alteration. Figure 6 shows the robustness of the hybrid interface when different levels of attenuation are applied to the validation set and the test set in Experiment 1. No statistically significant differences (Kruskal-Wallis test, *p* > 0.05) were found between the performance achieved when the same attenuation is applied to both set (white bars), or when the levels of attenuation differ of +10% (dark gray bars) or −10% (light gray bars).

**Table 1:** Classification performance averaged across subjects in five different conditions (100, 90, 50, 30, and 10% of EMG signal amplitude) using only the EMG signals or by the use of EEG and EMG signals together.

	**100% EMG**	**90% EMG**	**50% EMG**	**30% EMG**	**10% EMG**
**Recall**					
EMG	**0.95 ±0.01**	**0.95 ±0.02**	0.88 ± 0.19	0.63 ± 0.31	0.39 ± 0.36
EEG + EMG	0.95 ± 0.02	**0.95 ±0.02**	**0.90 ±0.09**	**0.79 ±0.17**	**0.66 ±0.21**
**Precision**					
EMG	**0.95 ±0.01**	**0.95 ±0.02**	0.87 ± 0.16	0.68 ± 0.21	0.36 ± 0.26
EEG + EMG	0.95 ± 0.02	**0.95 ±0.02**	**0.89 ±0.08**	**0.80 ±0.18**	**0.62 ±0.19**
**F1-Score**					
EMG	**0.95 ±0.01**	**0.95 ±0.01**	0.88 ± 0.16	0.58 ± 0.23	0.36 ± 0.26
EEG + EMG	0.95 ± 0.02	0.95 ± 0.02	**0.90 ±0.09**	**0.77 ±0.17**	**0.56 ±0.19**

*Performance are evaluated in terms of recall, precision, and F1-score. Bold values highlight the best classification approach for each condition*.

**Figure 7:**
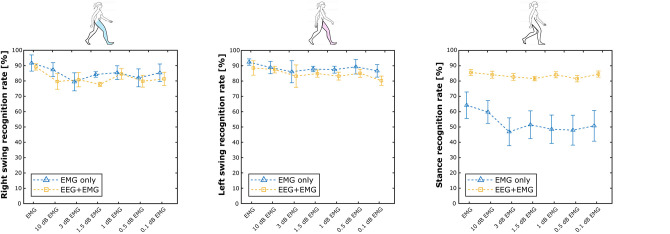
Average results achieved by the hybrid approach (yellow lines), with respect to the EMG only approach (blue lines), is shown at different levels of signal-to-noise ratio (in dB) of the EMG signals, as right swing (left plot), left swing (mid plot), and stance (right plot) phases recognition rate.

Table 2 summarizes the results of the Experiment 2. Overall, the hybrid approach improved the classification performance in all the experimental conditions, with accuracy and precision always above 80% on average. Figure 7 shows the performance scores for each gait event and for different SNR levels of the EMG signals. No statistically significant impact of classifier type and SNR level has been found for the right swing and the left swing recognition rate (Friedman test, *p* > 0.05), with more than 80% of correctly classified samples on average in all the conditions for both the EMG network and the hybrid approach. Differently, a significant improvement in the stance recognition rate became apparent with the fusion of EEG and EMG signals (Friedman test, *p* < 0.001), with the proposed hHMI keeping stable performance above 80%, regardless of the SNR level.

**Table 2:** Classification performance averaged across subjects at different levels of signal-to-noise ratio (in dB) of the EMG signal using only the EMG signals or by the use of EEG and EMG signals together.

	**EMG**	**10dB EMG**	**3dB EMG**	**1.5dB EMG**	**1dB EMG**	**0.5dB EMG**	**0.1dB EMG**
**Recall**							
EMG	0.83 ± 0.15	0.79 ± 0.16	0.71 ± 0.22	0.76 ± 0.13	0.74 ± 0.15	0.74 ± 0.20	0.75 ± 0.17
EEG + EMG	**0.89 ±0.06**	**0.86 ±0.08**	**0.82 ±0.13**	**0.85 ±0.07**	**0.84 ±0.08**	**0.84 ±0.09**	**0.83 ±0.8**
							
**Precision**							
EMG	0.81 ± 0.13	0.79 ± 0.14	0.70 ± 0.20	0.76 ± 0.12	0.74 ± 0.12	0.72 ± 0.17	0.76 ± 0.17
EEG + EMG	**0.88 ±0.06**	**0.85 ±0.08**	**0.80 ±0.11**	**0.83 ±0.07**	**0.83 ±0.06**	**0.82 ±0.07**	**0.82 ±0.09**
							
**F1-Score**							
EMG	0.79 ± 0.15	0.75 ± 0.14	0.66 ± 0.21	0.71 ± 0.14	0.71 ± 0.15	0.69 ± 0.19	0.73 ± 0.18
EEG + EMG	**0.89 ±0.06**	**0.86 ±0.07**	**0.80 ±0.12**	**0.84 ±0.06**	**0.82 ±0.07**	**0.83 ±0.08**	**0.82 ±0.08**

*Performance are evaluated in terms of recall, precision, and F1-score. Bold values highlight the best classification approach for each condition*.

## 4. Discussion

In this study, we propose a hybrid approach based on the fusion of brain and muscle activity exploiting the principles of Bayesian inference with the purpose of decoding gait events. In particular, we show the benefit of coupling a BCI and a myoelectric interface in order to cope with impairments affecting the reliability of muscular signals, either temporary (e.g., muscle fatigue) or permanent (e.g., muscle weakness). Indeed, from our experiments, the proposed hHMI achieved significantly better results than the single-signal EMG approach, particularly at higher levels of muscular signals degradation. Nevertheless, it is worth noting that the introduction of the EEG network did not affect the performance of the interface even if no alteration of the EMG signal was applied. Indeed, the EEG network achieved almost 90% of accuracy in discriminating between swing and stance phases, independently from the experimental condition, similarly to what has been obtained in Tortora et al. (2020).

The detection of walking patterns with the EEG signal alone is, not surprisingly, less accurate with respect to the results obtained using the EMG signal. Likely, while the location of the EMG channels provides a clear indication of which leg the subject intends to move, the localization of brain activity related to each leg has been always very challenging in BCI, due to their representation over the sensorimotor cortex very close to the deep interhemispheric fissure (Hashimoto and Ushiba, 2013). Despite this, many studies on MoBI frameworks have provided interesting evidence of consistent brain signal modulations correlated with walking patterns (Gwin et al., 2011; Cevallos et al., 2015; Storzer et al., 2016), revealing a direct connection between brain areas and lower limb muscles during locomotion (Artoni et al., 2017). For these reasons, in this study we decided to limit the output of the EEG network in recognizing stance and swing phases, regardless of the leg performing the step. Nevertheless, our results show that the capability of discriminating between swing and stance phases is what is most affected by the degradation of muscular information, as it can be seen by the performance of the EMG network for both experiments (Figures 5B, 7—right plot). On the other hand, the detection of right and left swing events remained stable over the different experimental conditions, particularly for the Experiment 2, with accuracy well over the chance level. In this sense, the proposed fusion approach is capable of integrating the decision of each classifier according to its estimated confidence in predicting each class. Thus, the decision making process is guided by the output of the most reliable interface for each class: the EMG network predicts the specific movement to be performed, i.e., a right or left step; the EEG network intervenes in the detection of intentional movement execution (i.e., swing) or not (i.e., stance), as suggested in literature (Leeb et al., 2011; Tonin et al., 2017; Tortora et al., 2019). Nevertheless, we suggest that the implementation of more advanced deep learning models including recurrent and convolutional layers (Tan et al., 2017; Ditthapron et al., 2019; Wilaiprasitporn et al., 2019) may help the discrimination of right and left leg movements, by integrating both time features in the low EEG bands [e.g., movement related cortical potentials (MRCP)] and frequency features in higher frequency bands (e.g., β-power modulation) (Sburlea et al., 2015; Storzer et al., 2016), enhancing the overall performance of our approach.

The physiological origin of muscle fatigue has been investigated in many studies (Enoka, 1992; Kos et al., 2008) and both increase and decrease of EMG activity have been found as a sign of fatigue (Gerdle et al., 2000; Dimitrova and Dimitrov, 2003). However, EMG increase is mostly present at the beginning of muscular fatigue occurrence as a compensatory strategy to keep the generated force constant. On the other hand, a strong decrease of EMG signals characterizes the steady-state condition of fatigue (Dimitrova and Dimitrov, 2003). From Figure 5A, the hybrid approach shows an effective contribution when the EMG amplitude drops below 50%, with significant differences at 30 and 10% of EMG signal amplitude. A precise condition in which we may find a muscle activity below 30% of its normal signal amplitude during walking it's hard to define from previous studies in literature, since normal gait analysis is compromised already at 40% loss of strength with respect to a physiological muscle activity (van der Krogt et al., 2012). Nevertheless, other biomechanical studies revealed a drop of more than 60% of the surface EMG amplitude after 60s of sustained isometric (Bigland-Ritchie et al., 1983) or repeated (Chesler and Durfee, 1997) contractions in healthy subjects. This behavior is accentuated in paraplegic patients when the muscle contractions are induced by FES in both upper limbs (Tepavac and Schwirtlich, 1997) and lower limbs (Mizrahi et al., 1994). In these cases, the EMG amplitude may drop below 20% of its initial amplitude after <1 min of FES. Although depending on the type of assistance provided and the severity of the disability, we suggest our hybrid interface to be effective if the walking task is sustained for several consecutive minutes or for several times during the day, which would be the case when these technologies are used in real-life applications. The online adaptation of a classifier to non-stationary changes of the input, such as those induced by muscle fatigue, is a current active research topic in human-machine interfaces, and in machine learning in general. One possible solution could be to train multiple models calibrated with EMG data at different level of muscle fatigue for each subject (Song et al., 2009; Artemiadis and Kyriakopoulos, 2010). However, these solutions come at the cost of a large training dataset required for the calibration of each model. Moreover, the reliability of the decision rule that determines when to switch the classification model may dramatically affect their performance. Differently, our approach does not require multiple models for the same signal, but to simply change the weights of the classifiers on the decision based on a gross estimation of the EMG signal reliability, for example by monitoring the median frequency of the EMG signal, that has been shown to be strongly correlated with muscle fatigue (Al-Mulla et al., 2009). Indeed, Figure 6 shows that the proposed hHMI provides robust performance even if the level of EMG alteration is not precisely known (Kruskal-Wallis test, *p* > 0.05).

In rehabilitation applications, it is expected for the motor functions of the patient to change as the therapy sessions progress, and even inside the same session, hopefully by progressively reducing the movement impairment and making the muscular activity more reliable. In this sense, the analysis of the performance of the hybrid classifier, contrary to the EMG only classifier, and the analysis of the parameters of the Bayesian fusion could provide a good quantitative assessment of motor functions improvement over time, guiding the therapy and the control strategy of the rehabilitation device.

While many papers may be found in literature on gait phase classification from EMG (Joshi et al., 2013; Luo et al., 2019; Morbidoni et al., 2019) with comparable results with respect to the EMG network used in this study (i.e., classification accuracy above 90% on healthy subjects), the EEG signals is rarely used to decode gait activity. Most of the studies in literature focused on the detection of walking initiation (Kilicarslan et al., 2013; Sburlea et al., 2015; Zhang et al., 2017) or on the offline analysis of the neural correlates of walking (Wagner et al., 2012; Storzer et al., 2016; Artoni et al., 2017). Another research group explored the use of EEG signals for the decoding of gait kinematics (Presacco et al., 2011; Nakagome et al., 2020), but not gait events. In our previous work (Tortora et al., 2020), we compared the results obtained with the EEG-driven LSTM network used in this study with respect to the methods proposed by Jorquera et al. (2013) and Shokur et al. (2018), that involved a similar classification problem, showing significantly better performance of our approach. On the other hand, a comparison of the performance of our hybrid approach with respect to other hybrid systems in literature is difficult since, as illustrated in the section 1.1, hybrid interfaces on lower limb applications are limited to classification scenarios that are very different from the one presented in this study. For all these reasons, we suggest our work to provide a strong contribution to the field of human-machine interface by proposing for the first time a hybrid approach for the purpose of decoding gait events from EEG and EMG signals.

A limitation of the approach described in this work is that it weights the classifier decision [i.e., *e*_*k*_ in (2)] only based on the performance estimated on the validation set (i.e., the confusion matrix), without considering the confidence the classifier had on that decision (i.e., the soft output of the softmax layer). As an example, considering a binary classification problem, the posterior probability (e.g., close to chance—0.6 vs. far from chance—0.9) can be considered as a measure of the confidence of the classifier. Thus, future works can investigate the implementation of a probabilistic fusion method to integrate the probability distribution of each classifier over the classes, so as to allow the decision making process to take into consideration both the uncertainty during validation and during online usage. Another limitation of this study is related to the dataset used for the evaluation of the hHMI performance, containing only data from healthy subjects walking over a treadmill, that will be accounted for in future studies by acquiring and testing this approach on real end users (e.g., stroke patients). Indeed, muscle weakness following paresis is a complicated phenomenon still under study, with many contributors, like loss of descending corticospinal pathway activation to motoneurons (Thomas et al., 1998), loss of large motor units (Lukács et al., 2008, 2009; Kallenberg and Hermens, 2009), disuse atrophy, fat infiltration (Ryan et al., 2011). Nevertheless, from studies on muscular activity and EMG-torque relationship, the EMG of muscles affected by weakness is principally characterized by an overall lower signal amplitude (Wang et al., 2013; Bhadane et al., 2016) and a worse SNR (Xu et al., 2012). Indeed, in paralyzed patients, the SNR can be <1.5 dB in the most severe cases, with respect to able-bodied subjects who are normally characterized by SNR of 20 dB or more (Wang et al., 2006). These evidences from literature support the validity of the applied permanent EMG alteration method during the Experiment 2, and the results we achieved suggest this approach to be promising to deal with lower limb paresis. Finally, future evaluations of this approach will be carried out during over-ground walking, to ensure that the same performance shown in this study are achieved when walking intention is purely self-controlled and not induced by an exogenous stimulation, e.g., treadmill.

## 5. Conclusions

This study implements for the first time a classification framework that simultaneously uses EEG and EMG signals in reconstructing the walking pattern (i.e., swing and stance phases of each leg), improving the results we previously achieved with an EEG-based LSTM network (Tortora et al., 2020). In neurorobotics applications, the possibility to have a robust online decoding of gait phases may be exploited to provide a continuous control input synchronized with the user's walking pattern, going beyond the simple detection of walking initiation to trigger the assistive device (Do et al., 2013; Kilicarslan et al., 2013; Liu et al., 2017). For example, the proposed interface may be used online to drive lower limb exoskeletons that implement different control strategies for swing and stance phases during locomotion (Kazerooni et al., 2006; Yeh et al., 2010; Villa-Parra et al., 2017). Moreover, BCI technologies are gathering a lot of attention also from an economic perspective, with new commercially available devices every year (Sawangjai et al., 2019). Thus, the development of software solutions enhancing their usability may greatly impact their distribution in both clinical and daily-living applications. Evidence from literature (Leeb et al., 2011; Müller-Putz et al., 2011; Carlson et al., 2013) if combined with our findings suggests that the use of hybrid human-machine interfaces, may be the key to develop reliable neurorobotics devices to assist or restore locomotion on a wider population of patients. In fact, they allow to integrate multiple BCI systems and multimodal information, like EEG and EMG signals, as well as information from robotic sensors (Lee et al., 2017; Beraldo et al., 2018a, 2019). In this context, the development of software ecosystems capable of integrating multimodal signals and assistive devices (Beraldo et al., 2018b; Tonin et al., 2019) may enhance the possibility for these technological solutions to be used outside a laboratory environment (Leeb et al., 2013).

## Data Availability Statement

The data analyzed in this study is subject to the following licenses/restrictions: data are available from corresponding author upon reasonable request. Requests to access these datasets should be directed to Stefano Tortora, tortora@dei.unipd.it.

## Ethics Statement

Ethical review and approval was not required for the study on human participants in accordance with the local legislation and institutional requirements. The patients/participants provided their written informed consent to participate in this study.

## Author Contributions

ST and FA analyzed the data and wrote the manuscript. CC and FA collected the data. SM, LT, and EM contributed to the interpretation of the results and writing of the manuscript. All authors discussed the results, reviewed and approved the final version of the manuscript.

## Conflict of Interest

The authors declare that the research was conducted in the absence of any commercial or financial relationships that could be construed as a potential conflict of interest.
